# Inhibition of the substantia nigra pars reticulata produces divergent effects on sensorimotor gating in rats and monkeys

**DOI:** 10.1038/s41598-018-27577-w

**Published:** 2018-06-19

**Authors:** Brittany L. Aguilar, Patrick A. Forcelli, Ludise Malkova

**Affiliations:** 1 Interdisciplinary Program in Neuroscience, Georgetown University, Washington DC, 20057 USA; 2 Department of Pharmacology & Physiology, Georgetown University, Washington DC, 20057 USA; 3 Department of Neuroscience, Georgetown University, Washington DC, 20057 USA

## Abstract

The basal ganglia are an evolutionarily old group of structures, with gross organization conserved across species. Despite this conservation, there is evidence suggesting that anatomical organization of a key output nucleus of the basal ganglia, the substantia nigra pars reticulata (SNpr), diverges across species. Nevertheless, there are relatively few comparative studies examining the impact of manipulations of SNpr across species. Here, we evaluated the role of SNpr in a highly conserved behavior: prepulse inhibition of the acoustic startle response (PPI). We performed parallel experiments in both rats and rhesus macaques using intracranial microinfusions of GABA_A_ agonist muscimol to investigate the role of SNpr in PPI. SNpr inactivation significantly disrupted PPI in rats, congruent with prior studies; however, in macaques, SNpr inactivation resulted in facilitation of PPI. We suggest that this difference in circuit function results from a divergence in anatomical connectivity, underscoring the importance of circuit dissection studies across species.

## Introduction

The acoustic startle response (ASR) is a well-defined whole-body reflexive contraction of skeletal and facial muscles in response to a sudden, intense stimulus^1–4^. This response is present across multiple species, and has been characterized behaviorally in rats, mice, guinea pigs, hamsters, non-human primates, and humans^5^. Across these species, startle responses may be modified through habituation, fear potentiation, pleasure attenuation, and prepulse inhibition (PPI). PPI is operationally defined as the reduction in magnitude of the acoustic startle response when a high-intensity startling stimulus (“pulse”) is preceded by a low-intensity stimulus (“prepulse”)^6^. It is an accepted measure of sensorimotor gating, conceptualized as the “ability to process selectively a continuous stream of sensory and cognitive information and to allocate selectively attentional resources to salient stimuli”^5^. PPI is of particular interest, as patients with schizophrenia, Parkinson’s disease, obsessive compulsive disorder, and Tourette syndrome all display deficits in this form of gating^7,8^. The stimuli and behavioral measures used across species are similar, making this behavior particularly amenable to comparative studies. While a great deal is known about the circuitry mediating PPI in rodents, little is known in primates. Moreover, while there have been reports of differing sensitivity to systemically administered drugs across strain and species^9–11^, circuit level manipulations have not been similarly evaluated.

An abundance of evidence from rodent studies suggests that the basal ganglia play a pivotal role in PPI. The basal ganglia are a shared feature of all vertebrate brains and receive cortical input terminating in the dorsal (caudate nucleus, putamen) and ventral (nucleus accumbens) striatum^12^. Both drug and lesion studies targeting nucleus accumbens, dorsal striatum^13–15^ and ventral pallidum have resulted in PPI deficits^16–23^. Primary output nuclei of the basal ganglia include the internal segment of the globus pallidus and substantia nigra pars reticulata (SNpr). Of these structures, the least is known regarding the role of the SNpr in PPI. One prior study reported deficits following lesions of the substantia nigra in rodents, however, the lesions employed damaged both the SNpr and the adjacent SNc^24^; no similar studies have been conducted in primates.

While the basic architecture of basal ganglia circuitry has been conserved across millennia of evolution, there is evidence to suggest that some connections have diverged across species^25,26^. In particular, while neurons of the rat SNpr are highly collateralized^27–29^ and target multiple output structures, neurons in the primate SNpr are segregated (with few collaterals reported) and typically project to only one target structure^26,30–32^. Comparative functional studies of this circuit remain sparse; in fact, despite decades of study in rodents, the functional topography of the SNpr has only recently been evaluated in the non-human primate^33^. Findings from our lab showed site-specific motor behaviors (e.g., dyskinesias, torticollis, circling behavior) evoked by transient inactivation of the primate SNpr^33^. This profile differed from that seen in rodents or cats, species in which only circling was reported^34–41^. The presence of these divergent behavioral profiles may be due to the different motor requirements and adaptations of each of these species and may be mediated by divergence in projections from SNpr^25–27,30^. The degree to which the role of the SNpr is conserved across species in other evolutionarily conserved behaviors remains to be examined; PPI is one such conserved behavior.

To determine the role of the primate SNpr, and directly compare it to the role of the rodent SNpr we microinjected the GABA_A_ receptor agonist muscimol into the SNpr of each species and evaluated PPI using equivalent task parameters. We observed that, in rodents, inactivation of SNpr resulted in a disruption of PPI, and in primates, inactivation of SNpr resulted in a facilitation of PPI. This finding, though surprising in the context of PPI conservation across species, may be explained through established anatomical differences in basal ganglia architecture.

## Results

### Verification of Infusion Sites in Non-Human Primate

**Figure 1:**
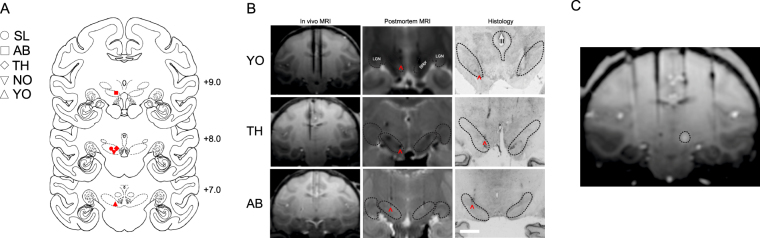
Localization of SNpr infusion sites in monkey. (**A**) Coronal planes from the public domain Rhesus Macaque “Red” Symmetrical Brain Atlas (Laboratory of Neuropsychology, National Insitute of Mental Health) showing SNpr (ventral dashed ellipses) as measured by distance from the interaural plane: anterior (+9.0 mm), central (+8.0 mm), and posterior (+7.0 mm). Solid symbols indicate infusion sites for each animal; dose of MUS administered was 9nmol per 1ul for all animals. The sites were reconstructed from *in vivo* MRI, postmortem MRI, or histology for all animals (SL, AB, TH, NO, and YO). (**B**) Three representative images (animals YO, TH, and AB) showing tungsten electrodes placed in SNpr during *in vivo* MRI, needle tracks indicating infusion sites in postmortem high-resolution MRI, and Nissl-stained histology. In the *in vivo* images, electrodes were placed dorsal to the SNpr and coordinates were adjusted prior to experimental drug infusions. Images correspond to their respective planes in A. The arrows point to the tips of cannula tracks in both postmortem and histological images. (**C**) MR image (animal YO) following infusion of gadolinium (5 mM, 1ul) into the medial SNpr. White area shows hypersignal (3 mm in diameter) indicating diffusion of the 1ul volume 60 min after infusion. MUS infused in this site in SNpr evoked motor response indicated in Table 1. LGN, lateral geniculate nucleus; SNpr, substantia nigra pars reticulata; III, third ventricle. The scale bar shown for the photomicrograph in Panel B, (Animal AB) = 5 mm. This scale bar also applies to the other photomicrographs in this figure.

Infusion site verification is shown in Fig. 1. The position of electrodes from *in vivo* MRI scans closely correspond to the localization of the cannulae tracks from postmortem MRI scans and histological reconstruction. Histological analysis confirmed localization of infusion cannulae to the *pars reticulata*, and not the immediately superior *pars compacta*. Representative MR images and photomicrographs are shown in Fig. 1B. Consistent with our prior reports, pharmacological inhibition of the SNpr by MUS infusion induced contraversive quadrupedal rotations and cervical dystonia (torticollis, head tilt). The presence of these behaviors and other nigra-evoked motor changes are shown in Table 1.

**Table 1:** Motor behaviors observed during unilateral SNpr inactivation with MUS in monkey.

Monkey	Injected Hemisphere (#)	Head Tilt	Contraversive Rotation	Body Lean	Leg Dyskinesia
TH	L (3)	+	+	+	+
NO	L (3)	−	+	−	−
TO	L (3)	++	++	+	−
AB	L (3)	++	++	nd	nd
SL	L (4)	+	++	−	−

++, behavior observed with short latency; +, behavior observed; −, behavior not observed; nd, not determined.

### Unilateral Infusion of Muscimol into SNpr potentiates PPI in Macaques

**Figure 2:**
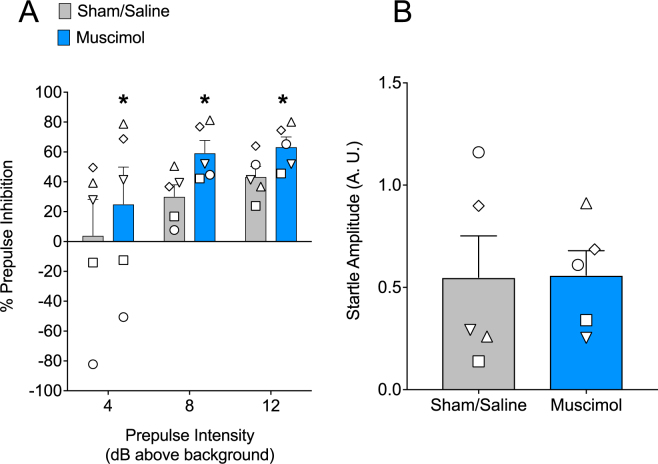
Prepulse inhibition is augmented by MUS in SNpr. (**A**) with no change to auditory startle response (**B**) in monkey. (**A**) Data show an increase in percent inhibition (+/− SEM) across prepulse intensities. The individual values for each animal are plotted as symbols that correspond to those plotted in Fig. 1. (**B**) Average of startle alone responses during PPI testing. * = PP4, P = 0.0046; PP8, P = 0.0009; PP12, P = 0.0046 (Holm-Sidak adjusted) compared with baseline.

To determine if unilateral pharmacological inhibition of SNpr would be sufficient to impair PPI in monkeys, we performed focal infusions of muscimol or saline into the SNpr of 5 macaques. The number of infusions per animal is shown in Table 1. Under control (saline-infused) conditions, animals displayed increasing PPI as prepulse magnitude increased (Fig. 2A). Muscimol (9nmol) infused unilaterally into the SNpr resulted in a significant increase in prepulse inhibition when compared within subject to a saline-infused baseline. A two-way ANOVA showed a main effect of drug treatment (F_1,4_ = 27.2, P = 0.006, η^2^_p_ = 0.947), while a trend toward an effect of prepulse intensity accounted for a substantial amount of the variability (F_2,8_ = 2.9, P = 0.1, η^2^_p_ = 0.899). No interaction was found between drug treatment and prepulse intensity (F_2,8_ = 1.1, P = 0.4, η^2^_p_ = 0.215). Holm-Sidak corrected planned comparisons revealed a significant increase in PPI following muscimol infusion at each of the tested prepulse intensities (Fig. 2A, PP4, P = 0.005; PP8, P = 0.0009; PP12, P = 0.005).

Baseline acoustic startle response (ASR), measured as the average response on pulse-alone trials, was not altered following muscimol infusion into SNpr (Fig. 2B, paired student’s t-test, t = 0.051, df = 4, P = 0.96, Cohen’s d = 0.023).

We also conducted bilateral infusions of MUS into the SNpr of 3 macaques (AB, YO, TH; 1–5 infusions per subject). One subject displayed severe motor incapacitation after bilateral injection and was not tested further (AB). Another subject (YO) displayed self-directed behavioral stereotypies and was thus injected only twice. Of note, when the two animals with repeated injections were analyzed (TH and YO) a similar profile of enhanced PPI was found under MUS-injected conditions (Supplemental Fig. 3); although it was not powered for statistical analysis.

### Histological Verification of Infusion Sites in Rodents

**Figure 3:**
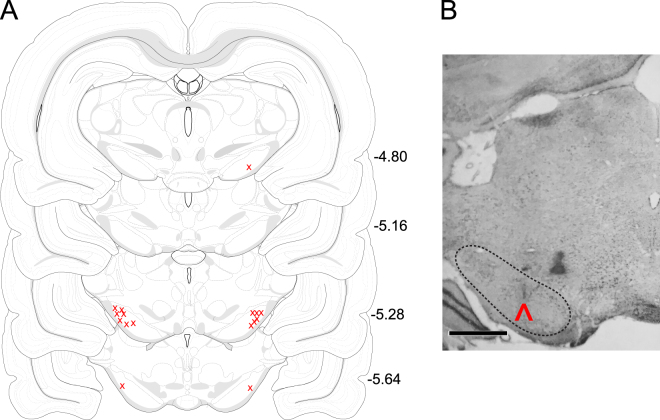
Localization of SNpr infusion sites in rat. (**A**) SNpr cannula placement. “X” indicates location of cannulae from animals used for data analysis. Data are plotted on planes from the BrainMaps 4.0 atlas^42^. This figure is not covered by the CC BY license, all rights reserved and used with permission of Dr. Larry Swanson. (**B**) Representative photomicrograph showing cannula placement in SNpr. Outline (dotted lines) shows anatomical boundaries. Arrowhead indicates cannula track. The scale bar in Panel B = 1 mm.

The position of cannula tips is indicated in Fig. 3A with representative photomicrographs showing localization of an injection site within SNpr (see Methods for further details). Data from Fig. 3A are plotted on open source Swanson Brain Atlas coronal slices^42^. Histological assessment was performed blinded to behavioral data.

### Unilateral or Bilateral Infusion of Muscimol into SNpr impairs PPI in Rodent

**Figure 4:**
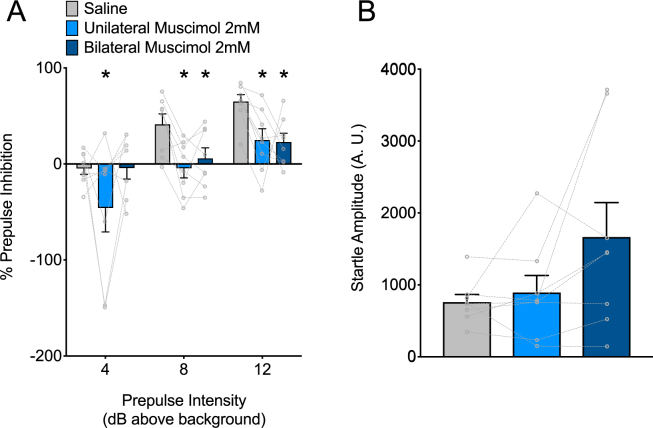
Infusion of MUS in SNpr impairs PPI in rat. (**A**) Prepulse inhibition as a function of prepulse intensity after infusion of saline (grey) or MUS unilaterally (light blue) and bilaterally (dark blue) in SNpr. * = significantly different than saline-infused control, Unilateral: PP4, P = 0.0183; PP8, P = 0.0087; PP12, P = 0.0156; Bilateral: PP4, P = 0.9786; PP8, P = 0.0230; PP12, P = 0.0156 (Holm-Sidak adjusted). (**B**) ASR after infusion of saline (grey) or MUS unilaterally (light blue) and bilaterally (dark blue) in SNpr. Individual points and lines indicate individual rat responses.

Of the 10 animals with correct cannula placement, 2 animals were excluded because they displayed no or negative prepulse values under control conditions (i.e., no drug present). The decision to remove these animals was made blind with respect to their performance under muscimol-infused conditions and was an *a priori* planned exclusion criterion that we have previously utilized in prepulse inhibition studies^21^. The data from the remaining 8 animals were used for further analyses. The effects of both unilateral and bilateral muscimol infusion into SNpr (1nmol muscimol) on PPI are shown in Fig. 4. Under control (saline-infused) conditions, PPI increased as a function of increasing prepulse intensity; this is consistent with prior reports^21,43^. Muscimol (1 nmol) infused unilaterally into the SNpr resulted in a significant decrease in prepulse inhibition at all prepulse intensities when compared within subject to a saline-infused baseline. Muscimol (1 nmol) infused bilaterally into the SNpr resulted in a significant decrease in prepulse inhibition at prepulse intensities of 8 and 12 above background when compared within subject to saline-infused baseline. A two-way ANOVA with drug and prepulse intensity as within subject variables yielded a main effect of drug (F_2,14_ = 5.7, P = 0.015, η^2^_p_ = 0.609), a main effect of prepulse intensity (F_2,14_ = 15.7, P = 0.0003, η^2^_p_ = 0.474) but no drug by prepulse intensity interaction (F_4,28_ = 1.6, P = 0.2, η^2^_p_ = 0.185). Pair-wise comparisons (Holm-Sidak adjusted) showed a significant difference between saline and muscimol infusion at each of the tested prepulse intensities for unilateral injection (PP4, P = 0.016; PP8, P = 0.009; PP12, P = 0.016). Bilateral infusions weren’t powered for analysis between saline and muscimol.

The effects of muscimol infusion in SNpr on baseline acoustic startle response (ASR) are shown in Fig. 4B. The pattern of response was equivalent to the monkey experiment. There was no significant change in baseline ASR under muscimol-infused conditions in either unilateral or bilateral infusions, however this trended toward statistical significance and infusion status accounted for 33.8% of the variability (one-way ANOVA comparing each group to saline, F_1.4,9.6_ = 3.6, P = 0.08, η^2^_p_ = 0.338).

## Discussion

Here, we report a striking difference in PPI responses following pharmacological inhibition of SNpr in rhesus macaques as compared to rats. Microinfusion of the GABA_A_ receptor agonist, muscimol, into the rodent SNpr resulted in a significant decrease in PPI of the acoustic startle response. By contrast, microinjection of muscimol significantly enhanced PPI in the monkey. These data underscore that despite the high degree of conservation of both this behavior and overall basal ganglia architecture, significant functional divergence also exists between these species.

Our findings in rats are consistent with a prior report using lesions^24^, despite two notable methodological differences from the present study. First, the excitotoxic lesions used by Koch and colleagues damaged both SN reticulata and compacta. In the present study, site targeting was restricted to SNpr as confirmed by both behavioral changes induced by drug administration and post-mortem histology. Second, we transiently suppressed activity in SNpr through focal drug infusion rather than permanent excitotoxic lesions. Transient inactivation offers the advantage of each subject acting as its own control and circumvents post-lesion neuronal plasticity. Given that our present findings are congruent with previously published data, we suggest that damage to SNpr was likely sufficient to account for the deficits Koch *et al*. observed.

It should be noted that several rodent studies have shown differences in drug effects between mice and rats, for example divergent effects of D1/D2 agonists across these species^9^ and strain- and dose-dependent effects of these drugs^44^. Additionally, one cross-species comparative study failed to translate ketamine-induced PPI deficits from rats to humans^45^. However, the present study is, to our knowledge, the first circuit-level species difference reported for prepulse inhibition.

Few studies have been published investigating PPI in monkeys; of those, most used systemic drug administration and were not focused on circuit-level manipulations^46–50^. The only prior study to investigate PPI circuitry in primates used excitotoxic lesions in cebus monkeys to evaluate the contribution of the superior colliculus^51^. Cebus monkeys are a New World monkey species, whereas the rhesus monkeys used in the present study are Old World species. In their experiment, Saletti *et al*. subjected 2 male cebus monkeys to ibotenic acid lesions of the superior colliculus; post-lesion sensorimotor gating experiments showed a significant decrease in PPI. They found that while there was no significant change in startle amplitude between the sham and lesioned animals, there was a significant reduction in PPI.

Similar to the findings in cebus monkeys, lesions of superior colliculus in rats reduces prepulse inhibition^52^. Activation of the superior colliculus, by contrast, produces the opposite response. Electrical stimulation of superior colliculus can serve as a prepulse to reduce acoustic startle responses^53^; similarly, focal blockade of GABA_A_ receptors in superior colliculus facilitates PPI^54^. Thus, prepulse inhibition of the acoustic startle response can be bidirectionally modified by activity within the superior colliculus. These findings may be of particular relevance to the present study as the major source of inhibitory input to the superior colliculus is the nigrotectal pathway, originating in substantia nigra pars reticulata^25,26,55,56^. Neurons in the SNpr display high tonic firing rates and pauses in the firing of SNpr neurons is associated with disinhibition of neurons in the superior colliculus^57,58^.

In isolation, one might expect that inhibition of the SNpr, which disinhibits superior colliculus, would result in a facilitation of PPI. However, both the prior lesion study in SNpr and our present findings using drug inactivation methods in rodents resulted in decreased PPI. Again, this differs from the primate, where inhibition of SNpr facilitated PPI. However, the nigrotectal pathway does not exist in isolation. Indeed, neurons in the SNpr also project to other targets, including parafascicular thalamus, globus pallidus internus (in rats the entopeduncular nucleus, EPN), pedunculopontine nucleus (PPN), and the superior colliculus^25,26,30,59^.

The effect of focal manipulation within brain regions that receive inhibitory input from the SNpr produces variable effects on PPI. For example, high frequency electrical stimulation of centromedian parafasicular complex results in enhanced PPI, as does deep brain stimulation (DBS) of EPN^60^. However, these studies were performed in rats selected for low PPI expression and the degree to which stimulation produced activation or depolarization block is unknown. The PPN, due to neurochemical heterogeneity, has been a difficult target for study. Non-selective lesions or focal injection of GABA agonist impair PPI^19,61,62^. Destruction of cholinergic neurons, which project to the caudal division of the pontine reticular nucleus (PnC), a well described obligatory relay station in the primary startle pathway, has produced either impairments or no effects depending on test parameters^63^. Interestingly, focal blockade of GABA_B_ receptors in PPN resulted in impaired PPI^62^. Thus, either excess inhibition or disinhibition of PPN can disrupt PPI. In contrast to the effects observed in other nigral target regions, increased activity within the deep and intermediate layers of the superior colliculus (DLSC) is associated with increased PPI, and decreased activity is associated with decreased PPI. Activation of DLSC may facilitate PPI through direct projections to raphe nucleus; activation of serotonin receptors within raphe nucleus has been shown to reverse amphetamine-induced PPI deficits^64^. Moreover, DLSC has direct projections to PnC^65–67^.

In rodents, inhibition of the SNpr is expected to modulate activity in all of nigral target regions, as neurons in SNpr are collateralized with projections from a single neuron terminating in thalamus, PPN, and DLSC^27,29^. In a study tracing SNpr projections, Cebrián *et al*. reported that 12 neurons out of 16 projecting to DLSC also projected to PPN^29^. Given the divergent effects each of these target regions has on PPI, the net result of inhibition of SNpr is unlikely to mirror the effects of activation of any individual efferent structure. This differs from the organization of SNpr in monkeys. In the non-human primate, discrete populations of cells within the SNpr project independently to each of these downstream targets, with minimal collateralization^25,26,30,68^. When comparing projections across species, Beckstead *et al*. reported co-labeling in SNpr after injection of retrograde tracers into DLSC and PPN; they reported 17% collateralization in cats and ~8% in monkeys, in contrast to the ~75% collateralization in rats reported by Cebrián *et al*. Although many of the critical anatomical findings were reported in New World monkeys (cebus)^25,30^, several groups have shown similar results in Old World monkeys^26,69^. Thus, there is a clear functional topography within the macaque SNpr that is not observed in non-primate species. A previous study from our lab demonstrated that inhibition of restricted sites within the central and medial SNpr produced cervical dystonia, an effect that was normalized by inhibition of DLSC^70^. Consistent with this, neurons within this region of the SNpr project to the DLSC^25,68^. Other sites within SNpr produced either quadrupedal rotations or limb dyskinesias^33^. Of note, rotational behavior and dyskinesias, but not cervical dystonia, can also be evoked by inhibition of GPi^71^. While GPi and SNpr share the majority of their projection targets, GPi does not project to the DLSC. Thus, our monkey injection sites targeting SNpr contain putative DLSC-projecting neurons. Microinfusion of muscimol into this population of SNpr neurons is expected to disinhibit DLSC but not PPN, which remains tonically inhibited by GABAergic projections from other regions of SNpr^30^. Consistent with this hypothesis, the animals in our study that showed short latency to cervical dystonia also displayed the greatest enhancement in PPI. While speculative, these differences in functional anatomy may provide an explanation for the opposing effects of SNpr inhibition on PPI across species.

These data provide the first direct comparison of circuit-level contributions to PPI across species. Despite the conservation of this reflex between rodents and monkeys, we observed a striking difference in the role of SNpr. These data underscore the importance of cross-species circuit characterization and caution against simple extrapolation from the rat to the primate, even for highly conserved behaviors.

## Materials and Methods

### Non-human Primate Studies

#### Experimental design and statistical analysis

To evaluate the role of SNpr in sensorimotor gating in macaques, we measured prepulse inhibition of the whole body acoustic startle response of monkeys seated in a primate chair. Manipulations were performed on a within-subject basis, with each animal receiving both saline and muscimol infusions. GraphPad Prism was used for data analysis and figure preparation. Prepulse inhibition was defined as [1 − (startle amplitude on prepulse trials/startle amplitude on pulse alone trials)] × 100 as calculated in previously published PPI studies^46,47^. Outliers were removed from data by performing a ROUT test (Q = 10%) individually on each session. Data were analyzed via a two-way analysis of variance (ANOVA) with prepulse intensity and drug treatments as within subject factors. The Greenhouse-Geisser correction for violations of sphericity was applied. Holm-Sidak corrections were applied to all planned comparisons to examine individual main and interaction effects. In addition to PPI analyses, we compared changes in ASR as a result of drug treatment. Mean ASR during pulse-alone trials was calculated for each animal during both saline and muscimol experiments and compared using a paired, two-tailed student’s t-test.

#### Subjects

Five, male, rhesus macaques (*Macaca mulatta*) were used in this study (TH, NO, AB, YO, SL). At the age of 2–3 years, they were procured from AlphaGenesis and transferred to Georgetown University, where all experimental procedures were conducted. Monkeys were pair-housed within two joined individual cages (size, 61 × 74 × 76 cm). They were raised in groups of three or four monkeys of the same age and were rotated through combinations of pairs within and across these three or four monkey groups over days and weeks. The monkeys were housed in a room with a regulated 12 h light/dark cycle and maintained on primate lab diet (Purina Mills, catalog #5049) supplemented with fresh fruit. Water was available ad libitum in the home cage.

Care and housing of the monkeys at the Georgetown University Research Resource Animal Facility met or exceeded the standards as stated in the Guide for Care and Use of Laboratory Animals (National Research Council (U.S.) Institute for Laboratory Animal Research, 2011), Institute for Laboratory Animal Research recommendations, and AAALAC International accreditation standards. The study was conducted under a protocol approved by the Institutional Animal Care and Use Committee at Georgetown University.

The present experiments began after the animals were extensively socialized and behaviorally trained (including chair training), and typically continued until the age of about 4 years. In addition to the experimental procedures described here, all subjects were trained on various cognitive tasks administered at the Wisconsin General Testing Apparatus; the tasks included the Hamilton Search task and an unconditioned fear task^72,73^. As part of those experiments, some animals received drug infusions in the BLA (all animals), deep and intermediate layers of the superior colliculus (DLSC; NO), parahippocampal cortex (PHC; SL, YO), and pulvinar (SL, YO, AB, TH).

#### Implantation of drug infusion platform and site verification

The monkeys were implanted with a stereotaxically positioned chronic infusion platform, which enabled us to target specific sites within the SNpr based on the coordinates assessed by structural magnetic resonance imaging (MRI) scans. For the preoperative and postoperative MRI and surgery, we followed the procedures as described in detail in our previous studies^74–76^. Briefly, before the surgery, each monkey received a T1- weighted MRI structural brain scan to calculate stereotaxic coordinates for the platform implantation (0.75 × 0.75 mm in-plane resolution, 1 mm slice thickness). The infusion platform was implanted under anesthesia and aseptic conditions^74,77^ followed by a postoperative regimen of analgesics and antibiotics determined in consultation with the facility veterinarian. Postoperatively, each monkey received at least one T1-weighted scan to obtain coordinates for infusions in the SNpr; tungsten micro-electrodes (FHC; Bowdoin, ME), which were visible on the scan, were used to determine the precise coordinates as described previously^70^. Fig. 1B shows the placement of a tungsten microelectrode at the dorsal boarder of the SNpr in three subjects.

#### Intracerebral drug infusions

To transiently inactivate the SNpr (for infusion sites, see Fig. 1A), 9 nmol of the GABA_A_ receptor agonist muscimol in a volume of 1 ul (MUS, 9 mM solution; Sigma-Aldrich) was infused at rate of 0.2 ul/min under aseptic conditions as previously described^78^. This dose proved to be effective to achieve rapid onset of both torticollis and quadrupedal rotational behavior in a prior study from our lab^33^. The entire infusion procedure lasted 10–15 min. At least 48 h elapsed between drug treatments in an individual subject. Each animal received at least 3 infusions of muscimol, and 3 control infusions. Control infusions consisted of either microinjection of an equivalent volume of sterile saline or a “sham” infusion. For sham infusions, all procedures were followed, but no cannula was lowered into place; sham infusions were included to minimize the number of brain penetrations.

We found no appreciable difference between sham and control infusions on a within subject basis, thus we collapsed these control infusions for further analyses. Sham infusions were performed in all animals; saline infusions were performed in YO, AB, and SL.

#### MRI verification of tissue volume reached by infusion

To verify the volume of diffusion of the infused solution, we infused 1ul (5nmol) of an MRI contrast agent, gadolinium (5 mM solution diluted in sterile saline; Magnevist; Fig. 1C). The range of diffusion visualized in MRI sections was limited to a diameter of 3 mm at 60 min after infusion, in agreement with previous gadolinium imaging in our laboratory and others^73,79^. This degree of spread is expected to cover the dorsoventral extent of SNpr.

#### Control behaviors

Prior to initiating PPI experiments, each injection site within the SNpr was examined for effects on motor behavior, as a positive control. Each animal was microinjected with muscimol (9 nmol, 1 ul) unilaterally into SNpr, videotaped, and scored for behavior. Each session was evaluated by a treatment-blind observer trained to detect each of the behavioral events (Noldus; Observer XT). The presence of the following events was recorded in freely moving animals: head tilt (torticollis, dystonia); quadrupedal rotations; body lean; and choreiform leg movements (dyskinesia). Descriptions of the behavioral events have been previously published^33^. Each behavior was scored for latency to onset of behavior and is presented in Table 1.

#### Prepulse inhibition (PPI) testing apparatus

Prepulse inhibition testing was conducted using an apparatus modified from that described by Winslow *et al*.^47^. Tests were conducted in a behavior room located next to the home cage room, in a sound attenuated chamber containing a primate chair (Crist Instruments Co.), which was attached to a platform sitting on a load cell (Med Associates). The chamber (60 × 114 × 80 cm) also contained a speaker (25 cm above the head) for administration of noise stimuli. The primates’ whole-body startle movements were transmitted via 50 kg load cell (Sentran LLC; YG6-B) located between the chamber floor and the primate chair platform. The load cell was connected to an amplifier which transmitted a signal to a Windows XP computer running the Startle Response software (Med Associates). Prior to beginning experimentation, we first calibrated the amplifier using a 10 KG weight and maintained this calibration setting across all animals. All animals weighed between 6–9 KG, so this single calibration was sufficient.

#### Optimization of prepulse and startle parameters

These tests were performed on 3 monkeys (NO, YO, and LO) and PPI tests were run on all animals. Two separate categories of startle response test were employed to optimize the startle response system prior to administration of the prepulse inhibition protocol. First, startle amplitude was measured in response to varying auditory stimuli intensities. Second, PPI protocols were optimized for ideal inter-stimulus interval (ISI). For these trials, ISI was manipulated (50–1040 msec) with other parameters held constant and tested using prepulses of 4 and 8 dB above background noise. A 50 ms (onset-to-onset) ISI was chosen for subsequent PPI studies as it produced the least variability between animals (Supplemental Fig. 1B).

Startle amplitude and optimized inter-stimulus interval (ISI) were measured in two separate sessions. Startle amplitude measurement sessions contained 10 equal and consecutive trial blocks of 4 white noise stimuli (blank, 90, 100, 105 dB) presented in a pseudorandom order. Inter-trial interval was a randomly selected value between 60–90 s and the duration of each stimulus was 40 ms. Background broadband noise with 70 dB intensity was administered for the duration of the testing period. Startle response was defined as the maximum peak-to-peak voltage amplitude of the load cell within the first 175 ms after stimulus presentation. Blank trials, during which no stimulus was presented but movement was recorded, were used as controls. Sound pressure levels were calibrated and verified using an SPL meter set to dB(A) weighting and with the microphone positioned at the level of the animals’ ear.

Once the optimal startle and prepulse parameters were determined, the pharmacological inactivation studies commenced.

#### Prepulse inhibition (PPI) protocol

Five animals were used in this experiment. 50 minute sessions consisted of a 3-min acclimation period with background noise (70 dB), 6 blocks of 3 randomized startling stimuli (90, 105, 110 dB; 40 ms pulse), 15 blocks of 4 randomized trials containing pulse-alone (105 dB; 40 ms) and prepulse-pulse (prepulses: 4, 8, and 12 dB above background noise; 20 ms) trials, and 10 blocks of 3 randomized startling stimuli (90, 105, 110 dB; 40 ms pulse). Pulse alone trials across the whole session were used as a control for maintenance of startle amplitude (Supplemental Fig. 1A) and blocks containing startling stimuli at the beginning and end were used to calculate habituation to startle. There was no significant change in startle response over the duration of the 15 trial blocks (one-way ANOVA, F_1.2, 3.7_ = 0.56, P = 0.5).

During the prepulse-pulse trials an inter-stimulus interval (onset to onset) of 50 ms was used. The inter-trial interval ranged from 15–30 seconds, randomly selected for each trial. Startle amplitude was defined as the peak load cell output voltage over a 175-msec period beginning at the onset of the pulse stimulus. Average startle response data are shown in Supplementary Figure 2.

#### Post-mortem MRI and Histology

Animals were perfused and, post-fixation, MR images were taken. For *post-mortem* MRI analysis, all available animals (TH, YO, AB, NO) were examined at high field strength (7 Tesla) on a Brucker Biospin Magnet using a Turbo-RARE pulse sequence, as previously described^73,74^. Following MR imaging, brains were processed for localization of infusion sites, as we have previously described^33,73,77^. Two animals (SL, NO) were unavailable for histological processing, but histological analysis was performed on animals TH, YO, and AB. Representative photomicrographs and MR images are presented in Fig. 1B.

### Rodent Studies

#### Experimental design and statistical analysis

To evaluate the role of SNpr in sensorimotor gating in rats, we measured prepulse inhibition of the whole body acoustic startle response of rats placed in a standard startle enclosure. Manipulations were performed on a within-subject basis, with each animal receiving both saline and muscimol infusions. GraphPad Prism was used for data analysis and figure preparation. Prepulse inhibition was defined as [1 − (startle amplitude on prepulse trials/startle amplitude on pulse alone trials)] × 100. Data were analyzed via a two-way analysis of variance (ANOVA) with prepulse intensity and drug treatments as within subject factors. Holm-Sidak corrections were applied to all planned comparisons. In addition to PPI analyses, we compared changes in ASR as a result of drug treatment. Mean ASR during pulse-alone trials was calculated for each animal during both saline and muscimol experiments and compared using a paired, two-tailed student’s t-test.

#### Subjects

Behavioral testing was conducted with 20 male Long Evans rats (Charles River) weighing approximately 180–200 g at the start of the study. Animals were housed in a temperature-controlled vivarium (22 C) at Georgetown University Medical Center and maintained on a standard 12 h light-dark cycle (lights on 0600–1800 h). All manipulations were performed in the light phase. All procedures were completed with approval from the Georgetown University Animal Care and Use Committee and in accordance with AALAC recommendations and the Guide for Care and Use of Laboratory Animals.

#### Implantation of drug infusion platform

Rats were anesthetized with equithesin (a combination of sodium pentobarbital, chloral hydrate, magnesium sulfate, ethanol, and propylene glycol) (2.5 ml/kg, i.p.). Animals were placed in a stereotaxic frame (Kopf, Tujunga, CA) for implantation of cannulae as previously described^21,80^. After shaving fur from the scalp, bupivicaine was injected at the site of incision (1.25 mg/kg, s.c.). Coordinates for the SNpr were derived from the atlas of Paxinos and Watson with animals positioned in the skull-flat plane^81^. Guide cannulae (22 gauge; Plastics One, Roanoke, VA) were fitted with 28-gauge internal cannulae that extended 1 mm beyond the tip of the guide, and implanted 4.92 mm posterior to bregma, 2.5 mm lateral to the midline, and 7.6 mm ventral to the dura. Cannulae were fixed to the skull with four jeweler’s screws using dental acrylic. 28-gauge dummy cannulae were inserted to maintain patency. Following surgery, rats were given carprofen (5 mg/kg, s.c.) as an analgesic, 1 ml warm normal saline (s.c.) to maintain hydration and placed on a heating pad to maintain body temperature until they fully recovered from anesthesia.

#### Intracerebral drug infusions

Muscimol (MUS, 9 mM solution; Sigma-Aldrich) was dissolved in saline to make a 2 mM solution, and infused at a dose of 1nmol in 0.5 ul per site; infusion procedure was as previously described^21^. In short, rats were infused (either unilaterally or bilaterally) with muscimol at a rate of 0.2 ul/min; cannulae were left in place for 1 minute after infusion to prevent spread of drug up into the cannula tract. Within the experiment the drug/saline infusions were counterbalanced, as were unilateral/bilateral injections. Animals received between 3–4 infusions.

#### Prepulse inhibition (PPI) testing apparatus

Testing occurred within three sound attenuated startle chambers (SR-Lab Startle Reflex System; San Diego Instruments, San Diego, CA) as previously described^21^. 10 minutes following intracerebral infusion, animals were placed into the startle chamber for behavior testing.

#### Prepulse inhibition (PPI) protocol

The PPI protocol was adapted from Forcelli *et al*.^21^ to match the primate protocol described in this paper. The 15 min sessions consisted of a 5-min acclimation period with background noise (70 dB), 5 habituating startling stimuli (105 dB; 40 ms pulse), 6 blocks of 4 randomized trials containing pulse-alone (105 dB; 40 ms) and prepulse-pulse (prepulses: 4, 8, and 12 dB above background noise; 20 ms). During the prepulse-pulse trials an inter-stimulus interval of 50 ms (onset to onset) was used. The inter-trial interval ranged from 15–30 seconds, randomly selected for each trial. Startle amplitude was defined as the peak piezoelectric accelerometer output over a 175-msec period beginning at the onset of the pulse stimulus. Sound pressure levels were calibrated and verified using an SPL meter set to dB(A) weighting and with the microphone positioned at the level of the animals’ ear.

#### Histology

Following the completion of behavioral testing, rats were overdosed with deep equithesin (4 ml/kg) anesthesia, perfused with ice-cold PBS and 4% paraformaldehyde, and decapitated. Brains were fixed in 4% paraformaldehyde for 24 hours to ensure complete penetrance, cryoprotected in 30% sucrose solution and frozen. Coronal brain sections (40 μm thick) were cut on a cryostat (Reichert Model 975 C) and stained with cresyl violet acetate. Microscopic examination was performed to verify the location of cannula injection sites in the SNpr according the atlas of Paxinos and Watson^81^. Data from Fig. 3A are plotted on open source Swanson Brain Atlas coronal slices^42^. A representative photomicrograph presented in Fig. 3B. Cannula localization was determined by two observers (BA and PAF), while blinded to the behavioral results for each animal. Infusion site verification resulted in the exclusion of the data from 10 animals in the SNpr group, with cannula tips falling outside of the boundaries of the SNpr. This higher than typical attrition rate was due primarily to “misses” early in the project, while the first author was still mastering stereotaxic surgery. Rather than exclude the first cohort of animals, we report the full group sizes for the sake of full transparency.

## Electronic supplementary material

Supplemental Figures
