# Supplementary material for: Inhibition of the substantia nigra pars reticulata produces divergent effects on sensorimotor gating in rats and monkeys
Source: Sci Rep. 2018 Jun 19;8:9369. doi: 10.1038/s41598-018-27577-w (PMC6008324; doi:10.1038/s41598-018-27577-w)

Title: Inhibition of the substantia nigra pars reticulata produces divergent effects on sensorimotor gating in rats and monkeys.

Authors: Brittany L Aguilar, Patrick A Forcelli, Ludise Malkova

Supplementary Figure 1. Psychophysics in monkey. ***A***, “Pulse Alone” was analyzed from Sham and Saline trials to determine stability of startle reflex over time. Data are from all monkeys included in PPI drug infusion studies. ***B***, PPI was measured at prepulse intensities of 4 and 8 above background noise to determine optimal inter-stimulus interval (ISI). 50ms onset-to-onset was chosen for PPI studies as it produced the least variability between animals. The individual values for each animal are plotted as symbols that correspond to those plotted in Figure 1. Hexagonal marker represents animal LO, a pilot animal that was not included in subsequent PPI studies.

Supplementary Figure 2. Average pulse response in monkey. AU response to the noise stimuli when they were delivered by themselves (Pulse), or following a prepulse (4, 8, 12). Data are from all monkeys included in PPI drug infusion studies.

Supplementary Figure 3. Prepulse inhibition is augmented by bilateral MUS in SNpr. Data show an increase in percent inhibition (+/- SEM) across prepulse intensities. The individual values for each animal are plotted as symbols that correspond to those plotted in Figure 1.

Supplementary Figure 1

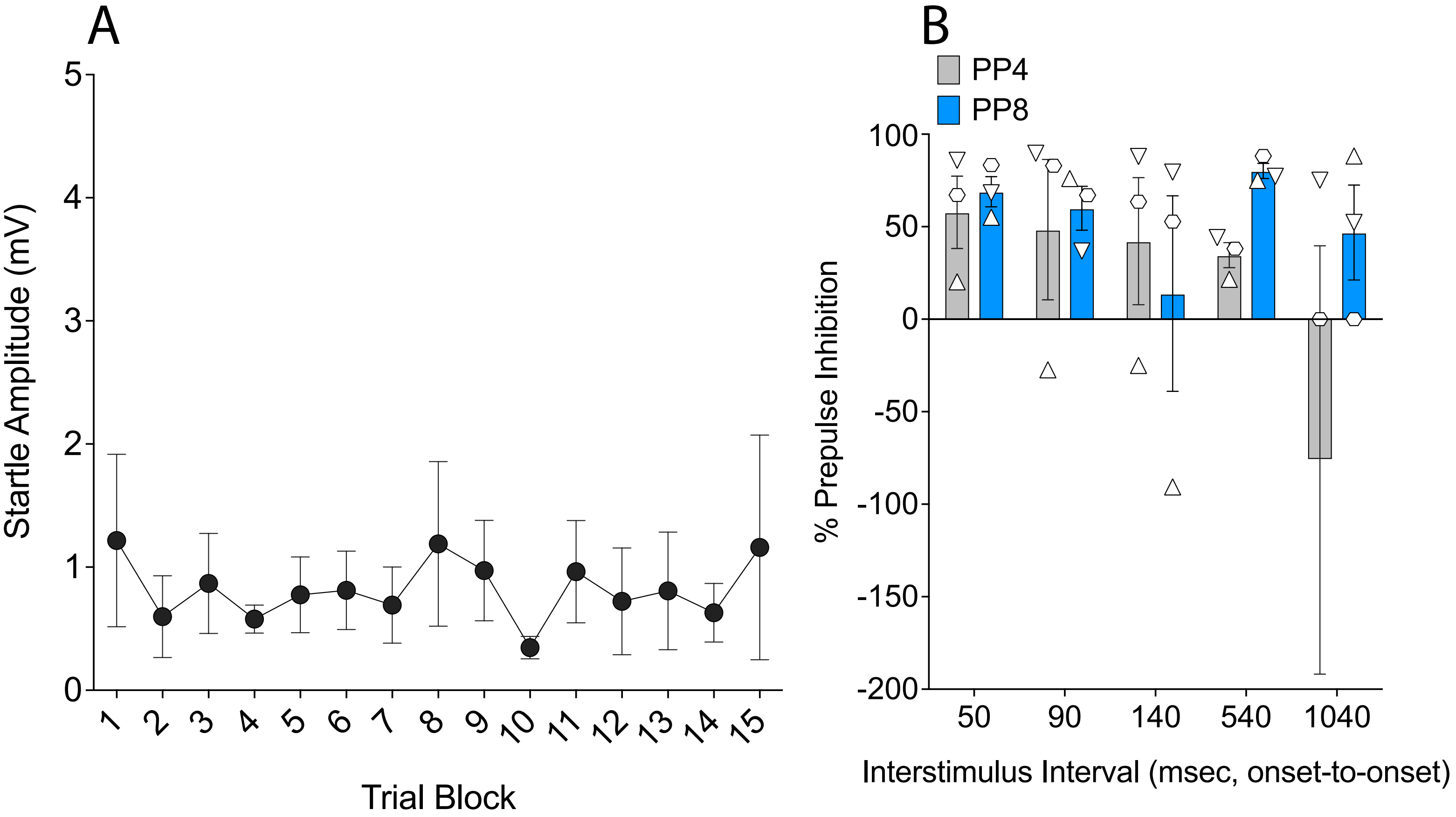

Supplementary Figure 2

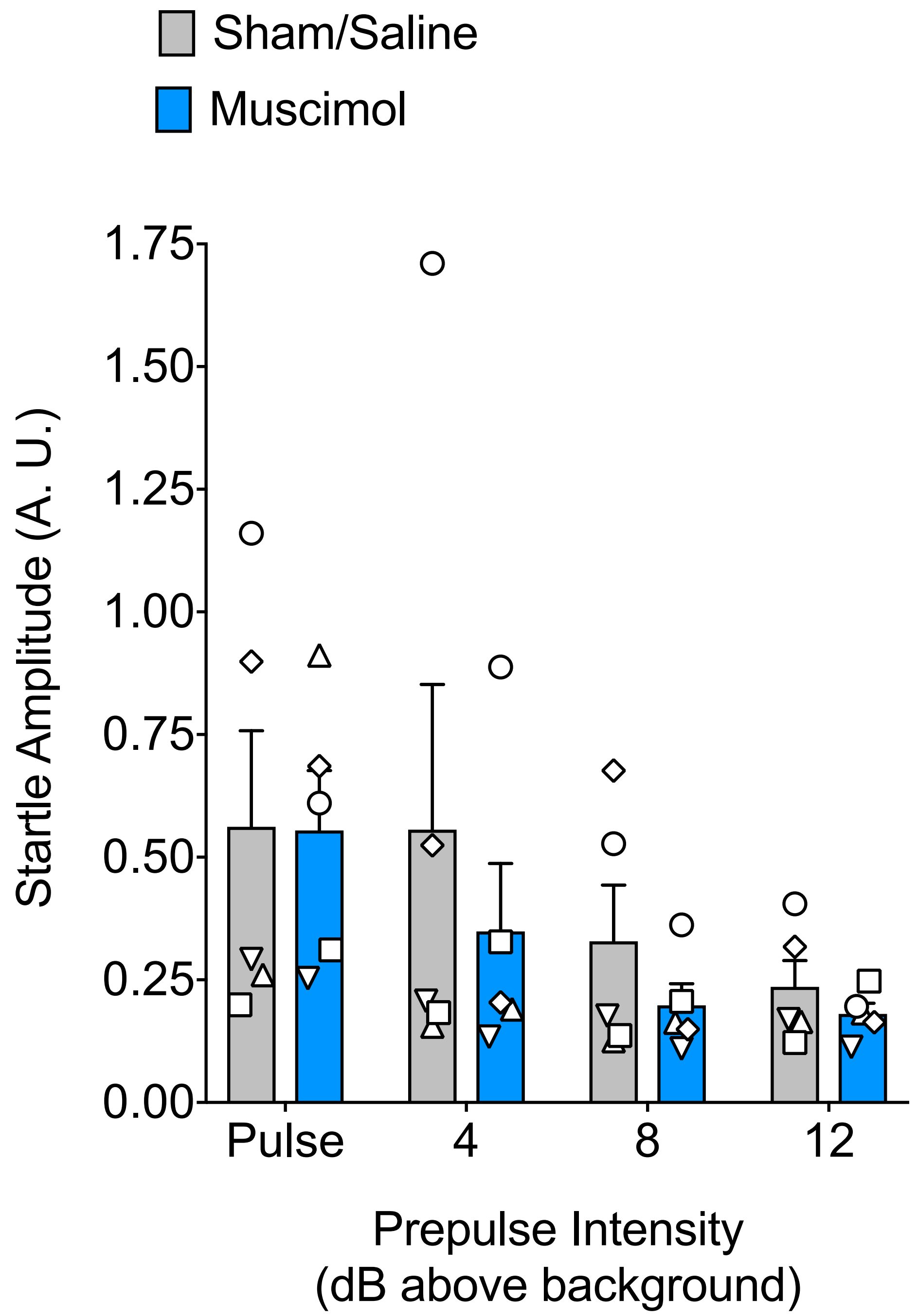

Supplementary Figure 3

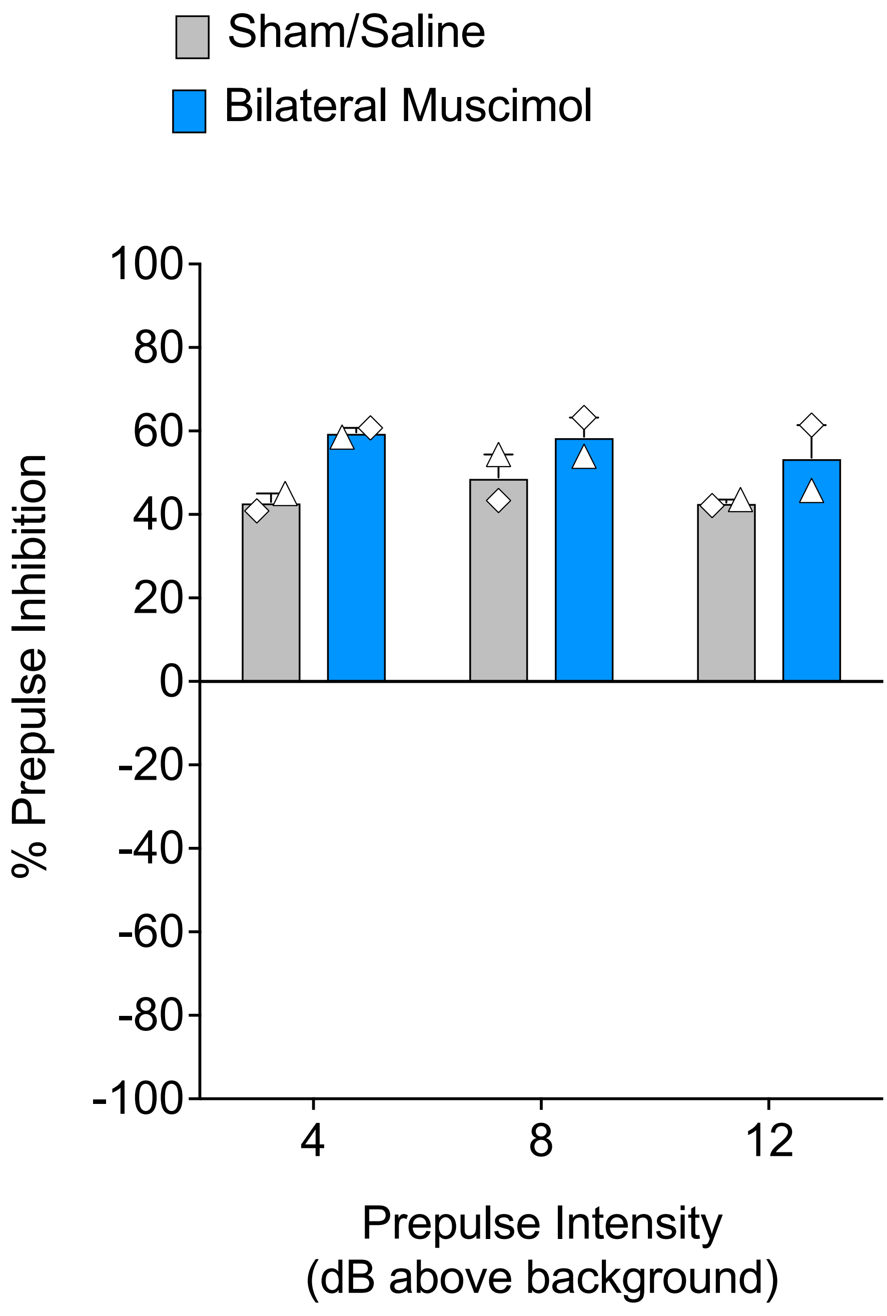

Supplement: Supplementary file 1 — Supplemental Figures [file 41598_2018_27577_MOESM1_ESM.pdf]
